# Mendelian Randomization and GWAS Meta Analysis Revealed the Risk-Increasing Effect of Schizophrenia on Cancers

**DOI:** 10.3390/biology11091345

**Published:** 2022-09-12

**Authors:** Kai Yuan, Weichen Song, Zhe Liu, Guan Ning Lin, Shunying Yu

**Affiliations:** 1 Shanghai Mental Health Center, Shanghai Jiao Tong University School of Medicine, Shanghai Jiao Tong University, Shanghai 200030, China; 2 Shanghai Key Laboratory of Psychotic Disorders, Shanghai 200030, China; 3 School of Biomedical Engineering, Shanghai Jiao Tong University, Shanghai 200030, China

**Keywords:** cancers, genetic susceptibility, genome-wide association study, mendelian randomization, schizophrenia, meta-analysis

## Abstract

**Simple Summary:**

The relationship between schizophrenia and tumors has sparked much interest and controversy. On the one hand, the health of patients with schizophrenia is affected by a variety of risk factors associated with cancer development, such as smoking, alcohol, and drug abuse. On the other hand, early investigations have found that patients with schizophrenia have a lower cancer incidence than the overall population. This phenomenon has prompted new theories on the processes underlying the protective effect. To explore the connection between schizophrenia and tumors, we used two-sample Mendelian randomization and GWAS meta-analysis methods on the GWAS summary data to assess potential genetic links between schizophrenia and 13 cancers. It was revealed that schizophrenia may lead to an increased risk of breast, ovarian, and thyroid cancers. Furthermore, the thyroid-stimulating hormone level is impacted by schizophrenia, which may further affect the estrogen and thyroid hormone levels. Meanwhile, based on our results, *AS3MT*, *SFXN2*, and *PCCB* may be potential biomarkers for preventing breast and thyroid cancers in patients diagnosed with schizophrenia. Therefore, we suggest that patients with schizophrenia should pay close attention to early risk warnings for breast, ovarian, and thyroid cancers.

**Abstract:**

The causal relationship between cancer and Schizophrenia (SCZ) remains controversial. Some researchers have found that SCZ is a cancer-preventive factor in cohort studies or meta-analyses, whereas others have found the opposite. To understand more about this issue, we used two-sample Mendelian randomization (2SMR) on available GWAS summary results to evaluate potential genetic connections between SCZ and 13 cancers. We discovered that the genetic susceptibility to schizophrenia lead to an increasing risk of breast cancer (odds ratio [OR] per log-odds increase in schizophrenia risk: 1.049, 95% confidence interval [CI]:1.023–1.075; *p* = 0.00012; FDR = 0.0017), ovarian cancer (OR, 1.326; 95% CI, 1.267–1.387; *p* = 0.0007; FDR = 0.0045), and thyroid cancer (OR, 1.575; 95% CI, 1.048–2.365; *p* = 0.0285; FDR = 0.123). Secondly, we performed a meta-analysis based on the GWAS summary statistics of SCZ and the three significant cancers. Next, we associated genetic variants to genes using two gene mapping strategies: (a) positional mapping based on genomic proximity and (b) expression quantitative trait loci (eQTL) mapping based on gene expression linkage across multiple tissues. As a result, we identified 114 shared loci and 437 shared genes in three groups, respectively. Functional enrichment analysis shows that the most enriched biological pathways are related to epigenetic modification. In addition, we noticed that SCZ would affect the level of thyroid-stimulating hormone (OR, 1.095; 95% CI, 1.006–1.191; *p* = 0.0354; FDR = 0.177), which may further affect the level of estrogen and the risk of the above three cancers. In conclusion, our findings under the 2SMR assumption provide crucial insights into the risk-increasing effect of SCZ on three cancers’ risk. Furthermore, these results may provide insights into understanding the genetic predisposition and underlying biological pathways of comorbid SCZ and cancers.

## 1. Introduction

The relationship between psychiatric disorders and tumors has sparked much interest and controversy [1]. On the one hand, the health of patients with Schizophrenia (SCZ) is affected by a multitude of risk factors associated with cancer development, such as smoking [2,3], alcohol [4], drug abuse [5], obesity [6], and physical inactivity [7]. Some epidemiological and meta-analyses have argued that SCZ can increase the risk of breast cancer [8,9]. On the other hand, early investigations have found that patients with SCZ have a lower cancer incidence than the overall population [10]. This phenomenon has prompted new theories on the processes underlying the protective effect [11]. Other studies and meta-analyses indicated that patients with SCZ have an approximately 50% increased risk of dying from cancer compared to the gender-age-matched healthy population [12,13].

Confirming a causal association between the two occurrences can be challenging in observational studies since the temporal sequence and influence of variables on exposure and outcome are impossible to identify. Large randomized controlled clinical trials are often regarded as the gold standard for determining causality, particularly when evaluating drug safety and efficacy [14]. However, because of the difficulty of implementation and the high cost of time and money, randomized controlled clinical trials may confront a variety of challenges when extrapolating to clinical practice.

Large-scale genome-wide association studies (GWAS) and Mendelian Randomization (MR) research have been widely used in recent years to investigate the causal relationship between complex exposure factors and disease outcomes due to better statistical methods and high-throughput sequencing technology [15]. To infer the relationship between phenotype and disease in MR, we used genotype as an instrumental variable (IV). For example, one MR research used anti-hypertensive drug target expression quantitative trait loci (eQTL) data with SCZ GWAS. Lower ACE mRNA and protein levels were found to be negatively linked with SCZ risk, indicating the importance of pharmacovigilance for ACE inhibitors in late-onset SCZ [16,17]. Several studies have now used the MR approach to investigate the association between SCZ and a single tumor (such as breast cancer or ovarian cancer), with the results indicating that there may be a causal relationship between them [18,19,20].

GWAS has identified a variety of genetic variants associated with complex diseases such as SCZ [21,22] and cancer [23,24], making it possible to perform MR analysis by using such genetic variants as IVs to detect potential causal associations of exposure with an outcome. This study adopted the two-sample MR (2SMR) method to identify potential evidence of a link between SCZ and 13 cancers. To discover more about the biological mechanism under this relationship, we merged cancer cis-eQTL data and immunohistochemistry staining images to identify appropriate genes to explore tumor biomarkers in patients with SCZ.

## 2. Materials and Methods

### 2.1. Conceptual Framework

**Figure 1:**
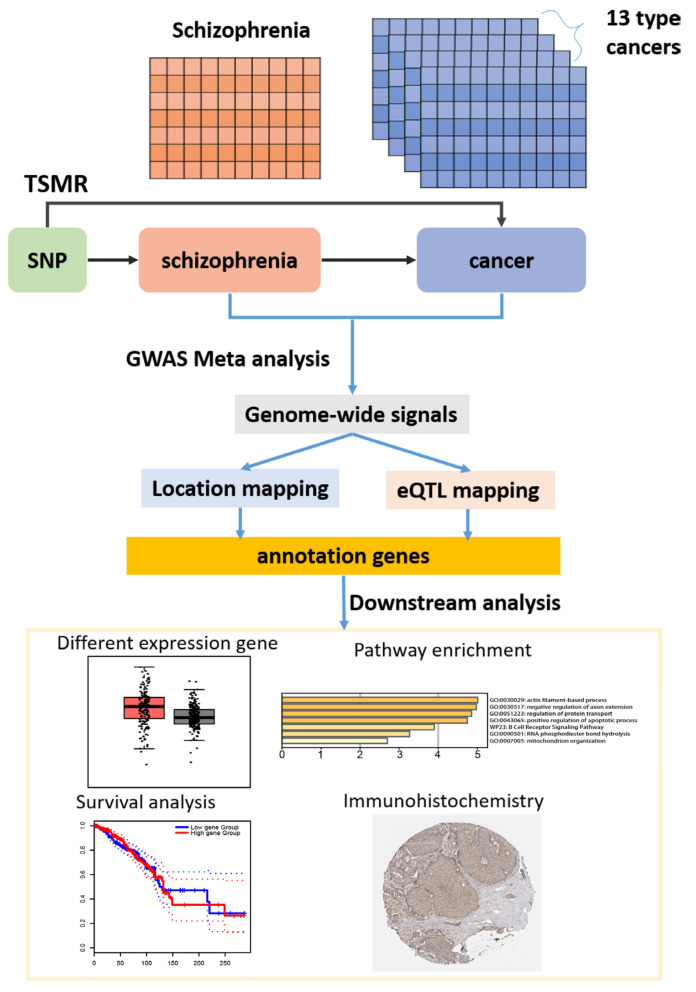
Flow chart that depicts the workflow of our study.

We employed 2SMR to examine the causative associations between SCZ and 13 types of cancers by single nucleotide polymorphisms (SNPs) using summary results from GWAS. Then using TCGA cis-eQTL data, we matched the SNPs to determine the eQTL genes and investigated their biological significance further (Figure 1). We expected to learn a lot about how genetic susceptibility to SCZ raises the risk of particular cancers from the 2SMR study. We conducted a meta-analysis based on the GWAS summary data of SCZ and three cancers to further explain the shared genetic risk loci for both diseases. Shared genetic variants with genes were connected using two gene mapping strategies: (a) location mapping based on genomic proximity and (b) eQTL mapping based on gene expression linkages across multiple tissues. The annotated genes investigated their biological significance further, and functional enrichment analysis was carried out. 

### 2.2. Data Sources

We used publicly available data from the cancers GWAS summary data from MRC Integrative Epidemiology Unit (IEU, https://gwas.mrcieu.ac.uk/, accessed on 14 August 2022), UK-bioBank (https://www.ukbiobank.ac.uk/, accessed on 14 August 2022) or BioBank Japan (BioBank Japan (biobankjp.org), accessed on 14 August 2022). Details of cancer data are shown in Tables S1 and S5. We accessed publicly available case-control GWAS summary data for SCZ (35476 cases and 46839 controls, 46 of European and 3 of East Asian ancestry) [22] and extracted the summary statistics for SNPs from their GWAS results strongly associated with SCZ (*p*-value < 5 × 10^−8^). The TCGA cis-eQTL data was downloaded through PancanQTL (http://bioinfo.life.hust.edu.cn/PancanQTL/, accessed on 14 August 2022) website [25].

### 2.3. Power Calculation

We calculated the variance in phenotype explained by each instrument by

(1) $$R^{2}=\frac{2*EAF*\left(1-EAF\right)*\beta^{2}}{2*EAF*\left(1-EAF\right)*\beta^{2}+2*EAF*\left(1-EAF\right)*N*se{\left(\beta \right)}^{2}}$$

where *EAF* was the effect allele frequency, *β* was the effect size, *N* was the sample size, and *se* (standard error, *β*) was the standard error of effect size. The F statistic was then denoted as

(2) $$F=\frac{R^{2}*\left(N-2\right)}{1-R^{2}}$$

*R*^2^ and F were used to evaluate the power for each instrument. MR power and the number of valid instruments for each pair are recorded in Table S2.

### 2.4. Two-Sample MR

Mendelian’s second law is the foundation of MR research. Alleles are randomly assigned when parents with two (or more) pairs of relative traits cross to produce gametes in their offspring [26]. For 2SMR analysis, the inverse variance weighting method (IVW) was utilized, which aggregated two or more random variables to minimize variance. It distinguishes itself by ignoring the intercept term and fitting with the reciprocal of the outcome variance as the weight. Thus, it can give reliable combined estimations of the effect of the exposure on the outcomes under conditions of heterogeneity between IVs, which may arise when quite a few IVs are included in an MR analysis [27]. In the IVW hypothesis, the IVs are not pleiotropic, considering that the results of GWAS are phenotypically standardized. Therefore, the results will be biased if these SNPs are pleiotropic when using the IVW method [28]. Correspondingly, there are ways to overcome the disadvantages of IVW, such as weighted median and MR-Egger. MR-Egger adds the intercept term, whose main purpose is to judge whether there is horizontal pleiotropic. Our study adopted 2SMR to explore the causality between SCZ and cancers using the “TwoSampleMR” package [29]. The effector alleles for each SNP were related to an elevated risk of SCZ when the GWAS data for SCZ was normalized. The effector alleles were then matched to the exposure dataset to create the appropriate SNP-cancer association dataset. Then, to calculate the 2SMR results, the IVW method was used to integrate the cancer factor-SNP and SCZ variable-SNP associated datasets, and the stats package in R was used to correct the FDR. Meanwhile, to ensure independence among genetic instruments, we applied Linkage disequilibrium clumping with a clumping window of 10 MB and an r2 cutoff of 0.001. For other parameters set as follows: the palindromes is 1, the align alleles is 1, and minor allele frequency threshold is 0.3.

### 2.5. Sensitivity Analyses

MR analysis also has certain restrictions. Firstly, the association hypothesis is that IVs are strongly associated with exposure factors, and weak IVs can lead to bias in estimates. Secondly, the independence hypothesis: IVs are not associated with confounding factors, such as population stratification. Finally, the exclusivity limitation: IVs are only associated with outcome variables generated by exposure factors [30]. Assuming that all conditions are satisfied, MR analysis can be used to overcome two major limitations of observational studies: unmeasured confounding and the inability to infer causality [31].

To assess whether the results were potentially biased, we used sensitivity analysis in MR analysis. Aiming at the three limitations of MR research, the following sensitivity analysis was designed:

#### 2.5.1. Heterogeneity Test

The primary purpose is to test the differences between IVs when there are non-specific SNPs (SNPs are related to the target exposure factors and other exposure factors). Heterogeneity analysis is needed to determine the influence of non-specific SNPs on the results. If the MR analysis results were still statistically significant after excluding non-specific SNPs, the evidence for a causal association between exposure factors and outcome variables would be verified. When we carried out the analysis, multiple SNPs were generally used as IV for causal inference, and it was difficult to avoid the influence of gene pleiotropy. Therefore, the MR-egger regression analysis method was needed to evaluate the bias caused by gene pleiotropy. The slope of the MR-egger regression direction could estimate the size of directional pleiotropy. MR-Egger was applied in each MR test, where heterogeneous SNPs were removed for all analyses. We reported the MR analysis results with them removed, and MR-Egger performed again after their removal to demonstrate whether heterogeneity existed.

#### 2.5.2. Pleiotropy Test

A fundamental assumption of MR is the exclusivity limitation, known as the ‘no horizontal pleiotropy’ assumption. Horizontal pleiotropy occurs when the variant affects other traits outside of the pathway of the exposure of interest and impacts the target outcome or when the variant directly affects the target outcome. As a violation of the ‘no horizontal pleiotropy’ assumption, horizontal pleiotropy can distort MR tests, leading to inaccurate causal estimates, loss of statistical power, and potential false-positive causal relationships. So the MR-PREESO package [32] was used to test horizontal pleiotropy.

#### 2.5.3. Leave-One-Out Sensitivity Test

It mainly calculates the remaining MR results of IV after removing IV one after the other. If the estimated MR results of other IVs after removing a certain IV differ significantly from the total result, it indicates that MR results are sensitive to that IV.

### 2.6. GWAS Meta-Analysis

Meta-analysis is becoming an increasingly important tool in GWAS of complex genetic diseases and traits [33]. Meta-analysis provides an efficient and practical strategy for detecting variants with modest effect sizes [34]. METAL provides a computationally efficient tool for meta-analysis of genome-wide association scans, which is a commonly used approach for improving power complex traits gene mapping studies. Meta-analysis of SCZ and cancer was performed with a sample size-based analytical strategy model using METAL [35], which is a fast and efficient meta-analysis of genomewide association scans. Specifically, METAL combines p-values across studies considering study-specific weights (the sample size) and direction of effect. METAL supplied the SNP ID, weight, alleles, frequency, effect size, standard error, and p-value from both GWAS summary statistics.

### 2.7. Identification of Candidate SNPs, Gene Mapping, and Functional Annotation

The tool FUMA [36] (v1.3.6) was used to find potential SNPs. Linkage disequilibrium blocks from 1000 Genomes Project Phase 3 [37] EUR population were used as a reference panel to compute r2 and MAF. Positional and eQTL mapping methods were used separately to map candidate SNPs to genes. Gene window for positional mapping was set at a default maximum distance of 10 kb on both sides and was based on ANNOVAR [38] annotation. Two sets of tissue types were employed for cis-eQTL mapping: (1) whole body tissues in GTEx v8 [39] (54 tissue types, including brain regions), and (2) 13 brain-only regions. SNPs were mapped to genes up to 1 Mb. Statistics were only applied to eQTLs with FDR 0.05. Genes that were mapped have their biotypes identified by Ensembl BioMart (Ensembl build v92, https://uswest.ensembl.org/info/data/biomart/index.html, accessed on 9 September 2022). Hypergeometric tests were used to perform functional enrichment studies. Information on pathways and functional gene sets was retrieved from MSigDB v7.0.

### 2.8. MAGMA Gene-Based Tests

The MAGMA [40] v1.08 with SNP-wise mean model as part of the FUMA pipeline was used to conduct the gene-based analysis. A 10 kb upstream and 10 kb downstream gene annotation window was used. 19,383 genes from Ensembl build v92 GRCh37 were assigned to SNPs. Based on GTEx v8 [39] RNA-Seq data, tissue expression (gene-property) analysis was carried out to evaluate the genetic connections of highly expressed genes in a particular tissue.

### 2.9. Immunohistochemistry

The protein expression of disease risk genes in cancers and normal tissues was analyzed by immunohistochemistry image in this study. The Human Protein Atlas (HPA) (https://www.proteinatlas.org/, accessed on 14 August 2022) is a freely accessible website that helps to study protein expression in human tissue and cells [41]. GeneMANIA (http://genemania.org, accessed on 14 August 2022) is a web tool for identifying internal associations of gene sets [42]. The interactions of common genes at the gene and protein expression level were identified with the help of GeneMANIA.

## 3. Results

### 3.1. SMR Results of SCZ and Cancers

**Figure 2:**
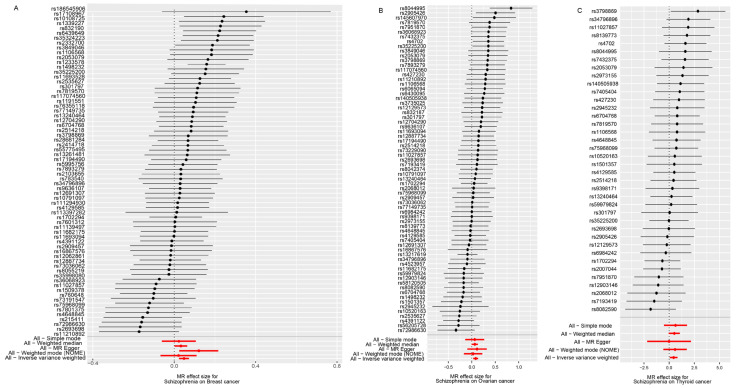
Estimated positive causal effects of genetically increased SCZ risk on three cancers risk. Forest plot representing the causal estimation of SCZ risk on (**A**) breast cancer, (**B**) ovarian cancer, and (**C**) thyroid cancer risk using each or all variants. Red lines are the causal effect of exposure on outcome is estimated using all SNPs using different methods.

Our findings revealed that SCZ was linked to an increased risk of breast cancer (odds ratio [OR] per one-standard-deviation increment of log odds of SCZ risk,1.049; 95% confidence interval [CI], 1.023–1.075; *p* = 0.00012; FDR = 0.00175; after removal of horizontally pleiotropic SNPs), ovarian cancer (OR, 1.326; 95% CI, 1.267–1.387; *p* = 0.0007; FDR = 0.0045) and thyroid cancer (OR, 1.575; 95% CI, 1.048–2.365; *p* = 0.0285; FDR = 0.123) (Table 1). MR-Egger was used to undertake various sensitivity analyses to uncover any violations of the third MR assumption. SCZ was found to have a moderately significant correlation with three cancers (breast cancer, ovarian cancer, and thyroid cancer) using the MR-Egger regression method (Figure 2A–C). Besides, no significant pleiotropy was identified in the MR-Egger pleiotropy test (*p*-value > 0.05) except for breast cancer (Table 1). Therefore, to reduce distorted effects of genetic IVs in breast cancer, eight horizontally pleiotropic SNPs (rs2905432, rs4702, rs58120505, rs11658257, rs73229090, rs4523957, rs11740474, rs12932476) were identified. The results of the leave-one-out analysis indicated that each variant had no significant effect on the overall IVW estimates, indicating that the SNPs utilized in this study were legitimate instruments (Figure 2A–C). The results for funnel plot asymmetry showed that the estimates of the precision (1/standard error) and Wald ratios for each SNP were bilaterally symmetrical (Figure 2A–C), confirming that SNPs used in this analysis were valid IV. We used another cancer data set for verification to ensure reliable results. In the European population data, we discovered that the probable causation between schizophrenia and breast cancer, ovarian cancer, and thyroid cancer remains considerable. Simultaneously, no significant association was observed when the 2SMR analysis was performed in an inverted direction, indicating that almost all cancers had no significant effect on SCZ risk (*p*-value > 0.05, Table S3), except the glioma (OR, 1.0559; 95% CI, 1.005–1.109; *p* = 0.030; FDR = 0.214).

### 3.2. SMR Results of SCZ and Subtypes of Three Cancers

The subtypes of each cancer were investigated further. Because breast cancer can be stratified by ER status, we employed MR to explore the impact of SCZ on ER-positive and ER-negative breast cancer risk. Using the random-effects IVW or the weighted median models, significant associations of genetically predicted SCZ risk with both ER-positive and ER-negative breast cancer risk were discovered, with ORs ranging from 1.03 to 1.05 (Table S4). Epithelial ovarian cancer can be categorized into five histological subgroups based on histology and molecular genetic alterations, including high-grade serous adenocarcinoma, endometriosis adenocarcinoma, clear cell adenocarcinoma, mucinous adenocarcinoma, and low-grade serous adenocarcinoma [43]. MR analysis were performed on these five types of ovarian cancer and SCZ is found to be significantly related to low-grade (OR, 1.235; 95% CI, 1.029–1.483; *p* = 0.024; FDR = 0.060) and high-grade serous adenocarcinoma (OR, 1.085; 95% CI, 1.015–1.160; *p* = 0.006; FDR = 0.03; after the removal of horizontally pleiotropic SNPs) (Table 2).

### 3.3. Shared Genetic Variants of SCZ with Three Cancers

Based on earlier GWAS research, we performed a genome-wide association meta-analysis of SCZ and three main cancers (Table 1). In the meta-analysis, we found 24,278 candidate related SNPs (*p* < 0.05) for 142 lead variants (*p* < 5 × 10^−8^) at 109 risk loci in SCZ and breast cancer. We found 465 lead variations (*p* < 5 × 10^−8^) at 227 risk loci, among 44,801 potential linked SNPs (*p* < 0.05), for ovarian cancer and 56,609 candidate related SNPs (*p* < 0.05) with 865 lead variations (*p* < 5 × 10^−8^) in 778 risk loci for thyroid cancer (Figure S1A–C).

**Figure 3:**
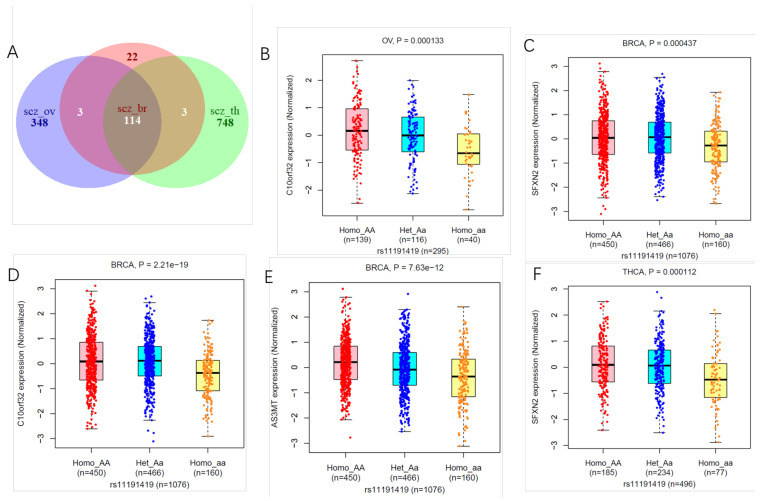
Shared variants regulated different genes expression in three tumors. Venn diagram showing (**A**) Number of specific and shared variants of three study cohorts; (**B**) Number of specific and shared genes of three study cohorts; (**B**–**F**) boxplot showing rs11191419 with eQTL genes expression in different cancers.scz_br: the common variants in schizophrenia and breast cancer; scz_ov: the common variants in schizophrenia and ovarian cancer; scz_th: the common variants in schizophrenia and thyroid cancer; OV: ovarian cancer; BRCA: breast cancer; THCA: thyroid cancer.

On the one hand, 114 SNPs appeared in all three sets of results (Figure 3A). According to annotation, the plenty loci were strongly correlated with Schizophrenia, especially rs11191419 (*p* < 5 × 10^−19^). It has been shown that the neural expression of *BORCS7*, *AS3MT,* and *NT5C2* is altered in SCZ due to genetic variation at rs11191419 [44]. These sites are significantly related to *AS3MT*, *SFXN2,* and *C10orf32* genes in three different tumors (Figure 3C–F). *AS3MT* is the main enzyme in the biomethylation of inorganic arsenic (iAs), a key enzyme that catalyzes the conversion of inorganic As2O3 to methylated products. *AS3MT* mRNA expression is closely related to the incidence of breast cancer [45,46]. Meanwhile, the protein expression level of AS3MT is tissue-specific and substantially expressed in the parathyroid and adrenal glands, according to the Human Protein Atlas database (HPA). Previous research indicated that *SFXN2* could further be manipulated in future experiments to diminish breast cancer tumors’ viability [47]. In breast and thyroid cancer, genes corresponding to the intersection sites (rs11191419, rs13240464, rs7432375) included *SFXN2*, *AS3MT, IMMP2L,* and *PCCB*. Using TCGA gene expression data, we found that the *AS3MT* and *PCCB* genes were differentially expressed in breast cancer, and the expressions of *SFXN2* and *PCCB* were significantly different in ovarian cancer (Figure S2A–D). Moreover, the expression of these genes was significantly correlated with the prognosis of breast cancer and thyroid cancer patients (Figure S2E,F). In the future, these genes could be employed as a biomarker to help SCZ patients avoid breast cancer and thyroid cancer. We evaluated immunohistochemical staining images from the HPA to learn more about AS3MT and SFXN2 protein expression in breast and thyroid cancer. The findings revealed that normal breast tissues had medium AS3MT protein expressions, whereas breast cancer tissues had low AS3MT protein expressions. Normal thyroid tissues had a medium level of SFXN2 protein expression, whereas thyroid cancer tissues had a high protein expression level. In conclusion, AS3MT protein expression was higher in breast cancer tissues than in normal breast tissues, and SFXN2 protein expression was higher in thyroid cancer tissues than in normal tissue (Figure S3A). We analyzed the relationship of common genes at the gene level using GeneMANIA (Figure S4B). The results showed that SLC5A6 was a Physical Interaction with PCCB and the diseases associated with SLC5A6 include Neurodegeneration, Infantile-Onset, Biotin-Responsive, and Biotin Deficiency. It can also be regarded as an important transporter for antipsychotic drugs [48]. Another important gene is SFXN1, which connects to SFXN2 by shared protein domains. SFXN1 is expressed in a number of cancer cells, with the highest expression in leukemia and lymphoma. Therefore, SFXN1 and its homologous proteins may be an important node regulating the fate of serine in cells and may also play an important role in tumor cell growth [49].

On the other hand, SCZ_ovrianrs-specific variants contain a large number of variants associated with breast cancer and ovarian cancer. rs10069690 polymorphism may be a risk factor for cancer, especially breast cancer, ovarian cancer, lung cancer, and thyroid cancer [50]. Other 14 SNPs (rs10069690, rs2853669, rs4702131, rs12519859, rs10941679, rs72765759, rs72749841, rs62355901, rs10472076, rs72774916, rs332529, rs6882649, rs6596100, rs11135046) were previously found primarily associated with risk of breast cancer [51]. For SCZ_thyroid-specific variants, we found the SNP rs965513 in 9q22, which has been consistently shown to be highly associated with increased papillary thyroid cancer risk [52].

### 3.4. Tissue Expression Specific and Gene Mapping

**Figure 4:**
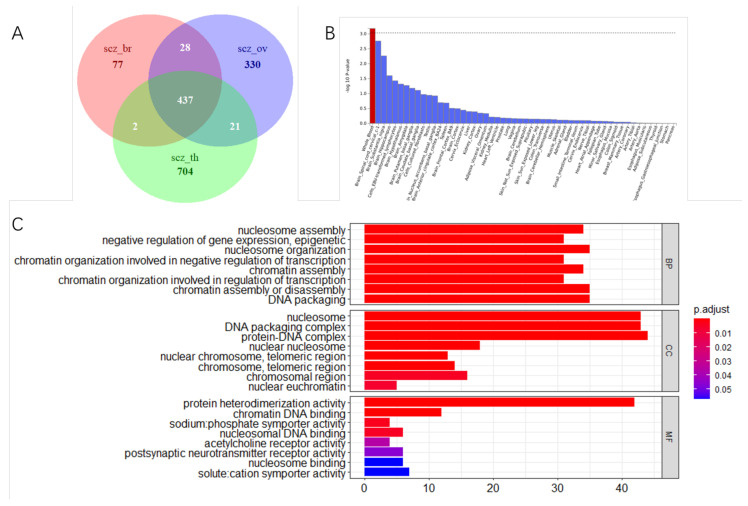
Functional enrichment of shared genes (**A**) Number of specific and shared genes of three study cohorts; (**B**) tissue expression enrichment in GTEx tissue types. The dashed line indicates the significance threshold at *p* < 0.001; (**C**) Bar-dot plots show significantly enriched gene sets annotation genes. scz_br: the common genes in schizophrenia and breast cancer; scz_ov: the common genes in schizophrenia and ovarian cancer; scz_th: the common genes in schizophrenia and thyroid cancer.

Here, we used two gene mapping approaches: (1) genomic proximity-based positional mapping and (2) eQTL mapping, which is based on linked gene expression across several tissues. As a result, 545 genes were shown to be associated with SCZ and breast cancer, 817 genes with ovarian cancer, and 1168 genes with thyroid cancer. Between the three gene sets, 437 genes are shared (Figure 4A). SNPs in the intronic region were followed by in the intergenic region, which contained the highest proportion of SNPs in the three study cohorts (Figure S4). Tissue enrichment analysis was performed using MAGMA to investigate the tissue expression of variant-associated genes. We found the SCZ_breast genes were majorly expressed in the whole_blood and brain spinal cord cervical (Figure 4B). The SCZ_ovarian genes were expressed highly in fibroblast cells and brain spinal cord cervical (Figure S4). Our results coincide with Li et al. [53]. We should associate these shared variations with genes in the genome to learn more about how they affect the underlying pathophysiology of SCZ and cancers.

Nucleosome assembly, epigenetic negative regulation of gene expression, and chromatin organization involved in negative regulation of transcription were the most notable enriched GO biological pathway gene sets for the shared genes. The most significant enriched Reactome gene sets for the SCZ and thyroid cancer-related special genes were HDACs deacetylate histones and hormone metabolic process pathways, which were enriched in GO biological processes. The top significant Reactome pathways using the SCZ and ovarian cancer-related special genes and the histone deacetylation pathway include deubiquitination and interleukin-7 signaling (Figure S5).

### 3.5. The Level of the Thyroid-Stimulating Hormone Could Be Affected by SCZ

It is generally known that female patients are more likely to develop breast, ovarian, and thyroid cancers and that these three diseases are closely linked to hormones in the human body (Figure S3C). As a result, we looked into whether SCZ is linked to hormones in a significant way. Previous research has suggested that thyroid hormone, estrogen, and other factors have a role in the pathogenesis of all three types of tumors. Thus, we performed a 2SMR study of SCZ, thyroid hormones, thyroid-stimulating hormones, and estrogen. The blood level of thyroid-stimulating hormone was found to be positively associated to the hereditary risk of SCZ (OR, 1.100; 95% CI, 1.011–1.197; *p* = 0.035; FDR = 0.177), while thyroid hormone receptor alpha (OR, 1.039; 95% CI, 0.952–1.134; *p* = 0.39), estrogen sulfotransferase (OR, 1.001; 95% CI, 0.923–1.086; *p* =0.976), estrogen receptor (OR, 0.963; 95% CI, 0.887–1.045; *p* = 0.363) and parathyroid hormone-related protein (OR, 1.000; 95% CI, 0.916–1.093; *p* = 0.992) did not change significantly (Table 3). Thyroid-stimulating hormone (TSH) is a hormone produced by the adenohypophysis that helps thyroid development and function. It encourages thyroid follicular epithelial cell proliferation, thyroid hormone synthesis, and release. TSH stimulates thyroid hormone production by acting on the thyroid gland. Thyrotropin can influence thyroid hormone and estrogen levels. As a result, it may develop associated tumors [54].

Since these three tumors were mainly adenocarcinomas, we performed MR analysis using different cancer types (adenocarcinoma, squamous cell carcinoma, large cell carcinoma). The results of the MR study of various cancer types revealed that squamous cell carcinoma had a substantial impact. SCZ was found to be related to squamous cell carcinoma but not to lung adenocarcinoma, squamous cell lung cancer, or basal cell carcinoma, according to the findings (Table S5). Such observation results may be related to the different data sources of squamous cell carcinoma and the collected patients’ self-reported database of squamous cell carcinoma. Furthermore, it is speculated that the genetic susceptibility of squamous cell carcinoma may function in different mechanisms compared with the above three solid tumors (breast, ovarian, and thyroid cancer).

## 4. Discussion

According to 2SMR, the analysis revealed that SCZ’s genetic susceptibility was linked to breast, ovarian, and thyroid cancer, and SCZ mainly affects high- and low-grade serous ovarian cancer. Based on meta-analysis and gene annotation, Numerous genes and loci detected may be intimately linked to variations in risk co-occurring in both diseases. We looked at the effect of genetic risk of SCZ on estrogen and thyroid-stimulating hormone and discovered that it primarily affected thyroid-stimulating hormone levels. Based on literatures [55,56], we believe that genetic susceptibility to SCZ may increase the expression of thyroid-stimulating hormone, which in turn increases the expression of thyroid hormone, thus resulting in patients with SCZ having an increased risk of breast cancer, ovarian cancer, and thyroid cancer.

Our study has several strengths. Firstly, this 2SMR analysis demonstrated a significant genetic influence of SCZ on breast, ovarian, and thyroid cancer risk using publicly available summary statistics from the largest-scale SCZ GWAS [22] and 13 types of cancers GWAS. Meanwhile, we eliminated pleiotropic SNPs that could have affected the results in MR studies. Besides, the IVW random-effects and weighted median model results suggest a reliable estimate. Second, Our meta-analysis was able to capture the significant signal from both SCZ and cancer summary statistics. We identified that *AS3MT* and *SFXN2* might be employed as biomarkers in the future to help SCZ patients avoid breast cancer and thyroid cancer. Finally, a hormone study demonstrated that the SCZ genetic locus might contribute to an increase in thyroid-stimulating hormone levels, which could influence the development of associated cancers.

Despite our findings showing an increased risk of three cancers in SCZ patients, there were some potential limitations in this study. Firstly, because SCZ represents a binary exposure in this 2SMR study, the random effects of IVW MR’s estimated influence on cancer risk may still be biased [57]. Second, breast and ovarian cancers are almost exclusively seen in women, and thyroid cancer is also more prevalent in women. As a result, demographic stratification may occur. Thirdly, the underlying biological processes of the increased risk of breast, ovarian, and thyroid cancers in SCZ patients are unknown and cannot be studied as outcome mediators such as drugs. For example, a previous study suggested that antipsychotic drugs may cause hyperprolactinemia and lead to breast cancer, but this conclusion cannot be confirmed for the time being. Because hyperprolactinemia is also present in patients with first-episode SCZ, some prolactin elevating antipsychotics have been shown to have a cancer-protective mechanism [58]. On the other hand, a meta-analysis of 16 cohort studies that included 480,356 schizophrenia patients and 41,999 cancer patients revealed that people with schizophrenia had a slightly significantly lower overall risk of cancer than the normal population [10], but this protective effect was not found in our results.

## 5. Conclusions

In conclusion, utilizing SCZ as an exposure factor and 13 cancers as an outcome, 2SMR analysis revealed that SCZ’s genetic susceptibility was linked to breast, ovarian, and thyroid cancer. It has been demonstrated using GWAS meta-analysis that epigenetic changes in Schizophrenia may create “molecular scars”, some of which may last the entirety of a person’s life and alter a person’s genetic susceptibility to other diseases. Future studies with individual genotype and phenotype data would be expected to determine the potential biological mechanisms underlying this causal relationship and promote tumor prevention and treatment in patients with SCZ.

## Figures and Tables

**Table 1:** The 2SMR results of SCZ and 13 cancers using IVW methods. Genetic susceptibility to SCZ may influence the risk of three cancers.

Datasets Num	Cancer Name	MR Results (IVW-*p* Value)	FDR	Heterogeneity Statistics	OR (95%CI)
ieu-a-1126	Breast cancer	**0.00012** *	0.0015	0.054	1.049 (1.023–1.075)
ieu-a-1120	Ovarian cancer	**0.0007** *	0.0045	0.2821	1.326 (1.267–1.387)
ieu-a-1082	Thyroid cancer	**0.0285** *	0.123	0.3841	1.575 (1.048–2.365)
ieu-a-822	Pancreatic cancer	0.1638	0.3549	0.7246	1.155 (0.943–1.415)
ieu-a-816	Neuroblastoma	0.504	0.655	0.6306	0.939 (0.782–1.128)
ieu-a-1057	Gallbladder cancer	0.843	0.843	0.095	0.876 (0.235–3.264)
ieu-a-1013	Glioma	0.225	0.417	0.274	0.880 (0.716–1.081)
ieu-a-966	Lung cancer	0.073	0.237	2.12 × 10^−34^	1.130 (0.989–1.290)
ieu-b-85	Prostate cancer	0.418	0.603	2.98 × 10^−15^	1.019 (0.973–1.068)
ieu-b-90	Oral cavity and pharyngeal cancer	0.681	0.804	0.00024	1.042 (0.858–1.265)
ukb-b-16713	Secondary malignant neoplasm of liver	0.158	0.332	0.4327	1.000 (0.999–1.0001)
ukb-b-20145	Colon cancer	0.812	0.879	0.8691	1.000 (0.999–1.0005)
ukb-b-19425	Rectum cancer	0.67	0.871	0.3638	0.9998 (0.9994–1.0003)

* Bold fonts represent the best experimental results. FDR: false discovery rate; Significance symbol conventions are *: *p* < 0.05.

**Table 2:** The 2SMR results of SCZ and five subtypes of ovarian cancers using IVW methods.

Datasets Num	Cancer Name	sample Size	MR results (IVW-*p* Value)	FDR	Heterogeneity Statistics	OR (95% CI)
Ieu-a-1125	Endometrioid ovarian cancer	43,751	0.8596	0.86	0.2153	1.0096 (0.908–1.122)
Ieu-a-1124	Clear cell ovarian cancer	42,307	0.1522	0.254	0.207	1.114 (0.961–1.291)
Ieu-a-1123	Invasive mucinous ovarian cancer	42,358	0.548	0.685	0.1659	1.046 (0.902–1.213)
ieu-a-1122	Low-grade serous ovarian cancer	41,953	**0.02374** *	0.06	0.101	1.235 (1.029–1.483)
ieu-a-1121	High-grade serous ovarian cancer	53,978	**0.006 (0.017)** *	0.03	0.1742	1.085 (1.015–1.160)

* Bold fonts represent the best experimental results. FDR: false discovery rate; Significance symbol conventions are *: *p* < 0.05.

**Table 3:** The 2SMR results of SCZ and hormones using IVW methods.

Datasets Num	Hormone Name	Samples Size	MR Results (IVW-*p* Value)	FDR	Heterogeneity Statistics	OR (95% CI)
prot-a-2974	thyroid hormone receptor alpha	3301	0.3866	0.644	0.1852	1.040(0.952–1.134)
prot-a-2892	estrogen sulfotransferase	3301	0.9762	0.992	0.5922	1.001 (0.923–1.086)
prot-a-991	estrogen receptor	3301	0.3628	0.644	0.7841	0.963 (0.887–1.045)
prot-a-530	thyroid-stimulating hormone	3301	**0.03536** *	0.177	0.3254	1.0999 (1.011–1.197)
prot-a-2432	parathyroid horm-related protein	3301	0.9921	0.992	0.1516	1.0004 (0.916–1.093)

* Bold fonts represent the best experimental results. FDR: false discovery rate; Significance symbol conventions are *: *p* < 0.05.

## Data Availability

All data analyzed in this study are curated from the public domain.

## Supplementary Materials

The following supporting information can be downloaded at: https://www.mdpi.com/article/10.3390/biology11091345/s1, Figure S1. Summary of snps and mapped genes for (a) SCZ_Breast cancer,(b) SCZ_ovarian cancer,(c) SCZ_thyroid cancer; Figure S2. The expression of mapping genes (A) *AS3MT* (B) *SFXN2* (C) *IMMP2L* (D)*PCCB* were differentially expressed significantly in cancers and significantly correlated with the prognosis of (E) breast cancer and (F) thyroid cancer patients; Figure S3. (A), Immunohistochemistry staining images from Human Protein Atlas showed the protein expression levels of AS3MT and SFXN2 in breast or thyroid cancers. (B), Gene-gene interaction network among common genes (*AS3MT, SFXN2, PCCB, IMMP2L*) in the GeneMANIA dataset. (C), possible explanations for observed association in 2SMR analysis between SCZ and cancer risk; Figure S4. Functional consequences of SNPs on genes for (a) SCZ_Breast cancer, (b) SCZ_ovarian cancer,(c) SCZ_thyroid cancer. tissue expression enrichment in GTEx tissue types for (D) SCZ_ovarian cancer,(E) SCZ_thyroid cancer; Figure S5. Pathway enrichment for (a) SCZ_Breast cancer, (b) SCZ_ovarian cancer,(c) SCZ_thyroid cancer related genes; Table S1. The information of 13 cancers data; Table S2. Summary statistics for all instrument variables after data harmonization; Table S3. 2SMR results of all cancers and SCZ; Table S4. 2SMR results of SCZ and 2 subtypes breast; Table S5. 2SMR results of SCZ and different type cancers.

## Abbreviations

SCZ	Schizophrenia
2SMR	Two-sample Mendelian randomization
GWAS	Genome-wide association studies
eQTL	Expression quantitative trait loci
SNP	Single nucleotide polymorphism
IVW	inverse-variance weighted method
MR	Mendelian randomization
MR-PRESSO	Mendelian randomization Pleiotropy RESidual Sum and Outlier
OR	odds ratio
IV	Instrumental variable;
CI	Confidence interval.
TSH	Thyroid-stimulating hormone
