# Supplementary material for: Mendelian Randomization and GWAS Meta Analysis Revealed the Risk-Increasing Effect of Schizophrenia on Cancers
Source: Biology (Basel). 2022 Sep 12;11(9):1345. doi: 10.3390/biology11091345 (PMC9495962; doi:10.3390/biology11091345)
Supplement: Supplementary file 1 [file biology-11-01345-s001.zip › Diagram legends.pdf]

# Diagram legends

**Figure S1.** Summary of snps and mapped genes for (a) SCZ_Breast cancer,(b) SCZ_ovarian cancer,(c) SCZ_thyroid cancer.

**Figure S2.** The expression of mapping genes (A) *AS3MT* (B) *SFXN2* (C) *IMMP2L* (D)*PCCB* were differentially expressed significantly in cancers and significantly correlated with the prognosis of (E) breast cancer and (F) thyroid cancer patients.

**Figure S3.** (A), Immunohistochemistry staining images from Human Protein Atlas showed the protein expression levels of *AS3MT* and *SFXN2* in breast or thyroid cancers. (B), Gene-gene interaction network among common genes (*AS3MT*, *SFXN2*, *PCCB*, *IMMP2L*) in the GeneMANIA dataset. (C), possible explanations for observed association in 2SMR analysis between SCZ and cancer risk.

**Figure S4.** Functional consequences of SNPs on genes for (a) SCZ_Breast cancer, (b) SCZ_ovarian cancer,(c) SCZ_thyroid cancer. tissue expression enrichment in GTEx tissue types for (D) SCZ_ovarian cancer,(E) SCZ_thyroid cancer.

**Figure S5.** Pathway enrichment for (a) SCZ_Breast cancer, (b) SCZ_ovarian cancer,(c) SCZ_thyroid cancer related genes.

**Table S1.** The information of 13 cancers data

**Table S2.** Summary statistics for all instrument variables after data harmonization.

**Table S3.** 2SMR results of all cancers and SCZ

**Table S4.** 2SMR results of SCZ and 2 subtypes breast

**Table S5.** 2SMR results of SCZ and different type cancers
