# Supplementary material for: Mendelian Randomization and GWAS Meta Analysis Revealed the Risk-Increasing Effect of Schizophrenia on Cancers
Source: Biology (Basel). 2022 Sep 12;11(9):1345. doi: 10.3390/biology11091345 (PMC9495962; doi:10.3390/biology11091345)
Supplement: Supplementary file 1 [file biology-11-01345-s001.zip › Figure S1.pdf]

A

#Genomic risk loci	109
#lead SNPs	142
#Ind. Sig. SNPs	413
#candidate SNPs	24278
#candidate GWAS tagged SNPs	11055
#mapped genes	545

B

#Genomic risk loci	227
#lead SNPs	465
#Ind. Sig. SNPs	1596
#candidate SNPs	44801
#candidate GWAS tagged SNPs	24111
#mapped genes	817

C

#Genomic risk loci	778
#lead SNPs	865
#Ind. Sig. SNPs	1076
#candidate SNPs	56609
#candidate GWAS tagged SNPs	7977
#mapped genes	1168
