# Supplementary figures and images for: Mendelian Randomization and GWAS Meta Analysis Revealed the Risk-Increasing Effect of Schizophrenia on Cancers
Source: Biology (Basel). 2022 Sep 12;11(9):1345. doi: 10.3390/biology11091345 (PMC9495962; doi:10.3390/biology11091345)

A

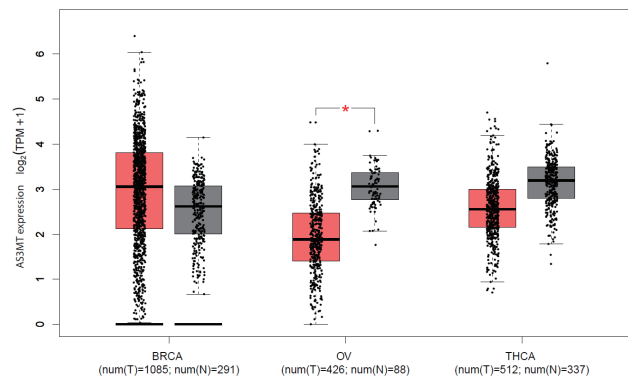

B

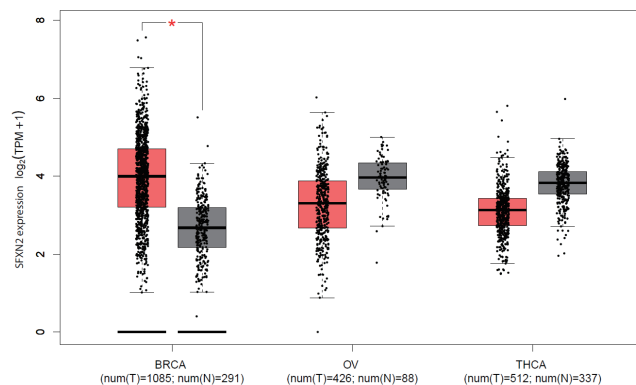

C

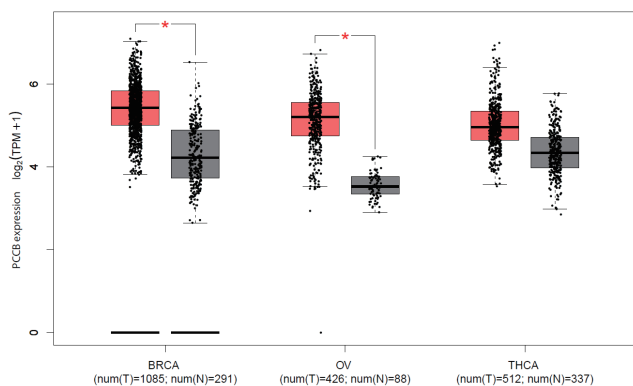

D

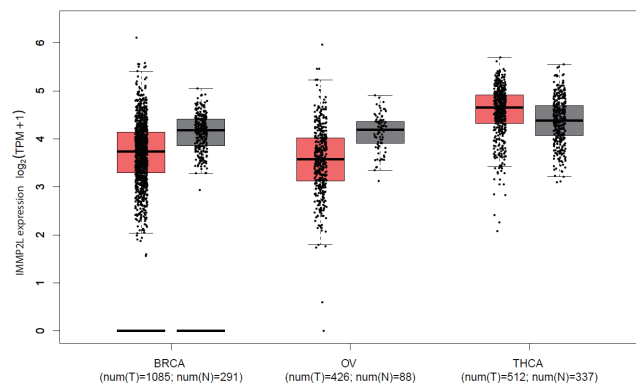

E

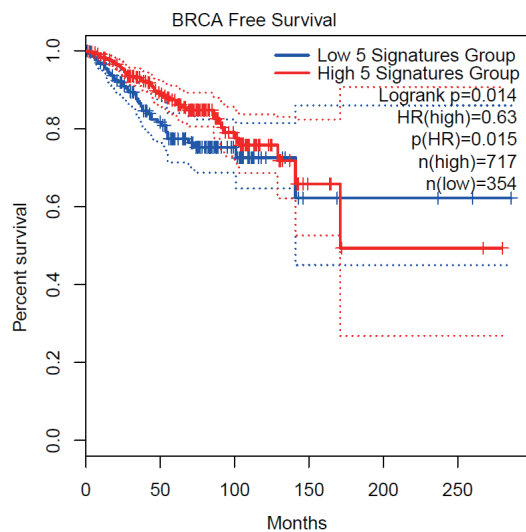

F

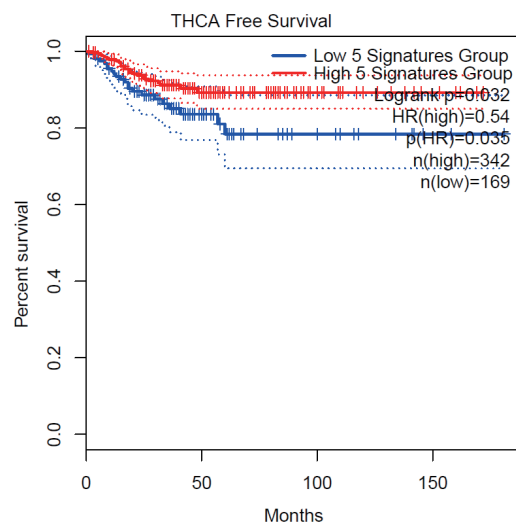

Supplement: Supplementary file 1 [file biology-11-01345-s001.zip › Figure S2.pdf]

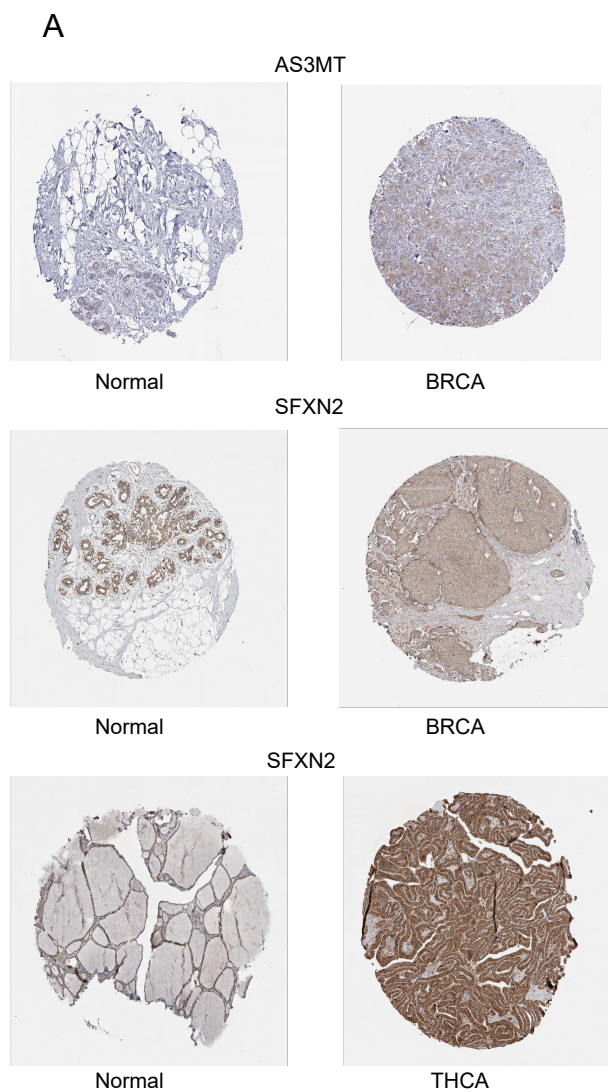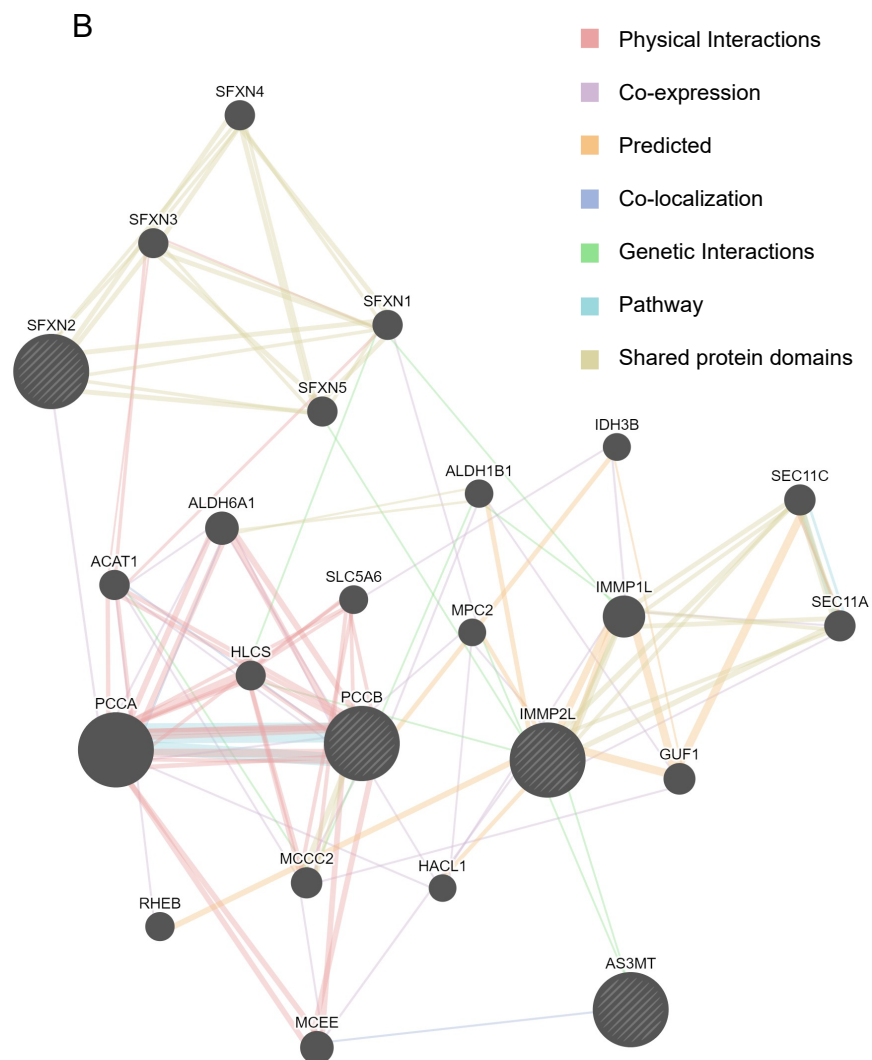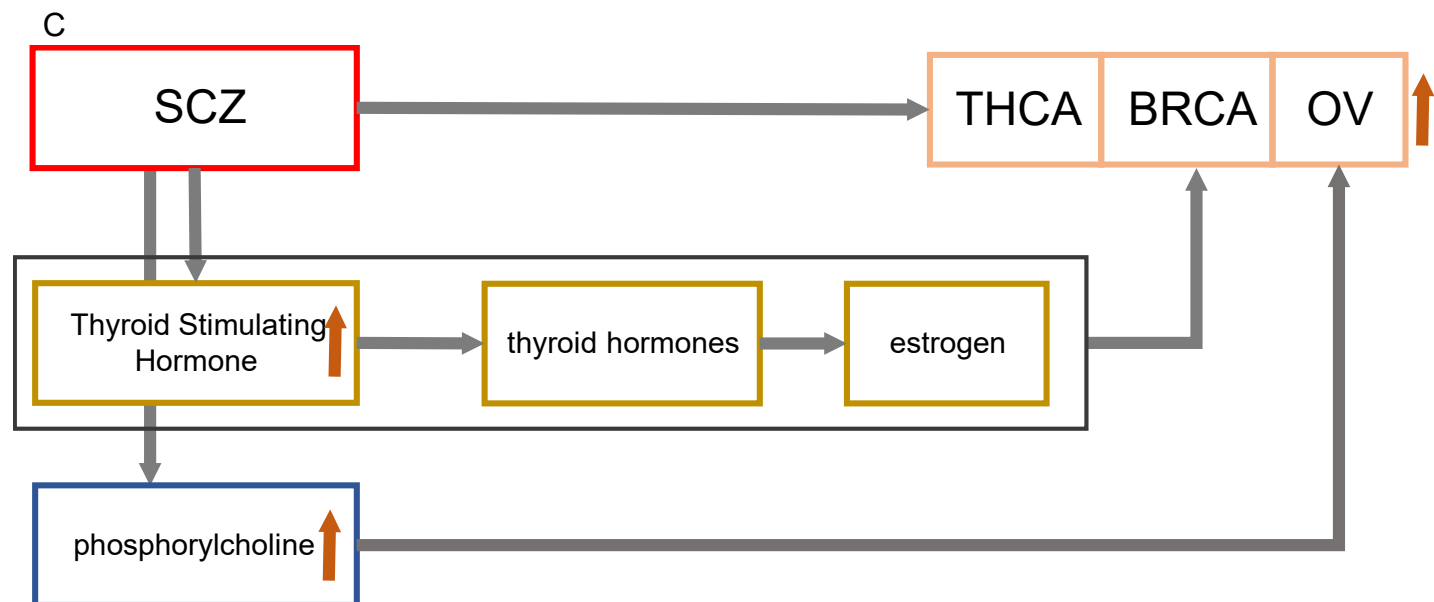

Supplement: Supplementary file 1 [file biology-11-01345-s001.zip › Figure S3.pdf]

A

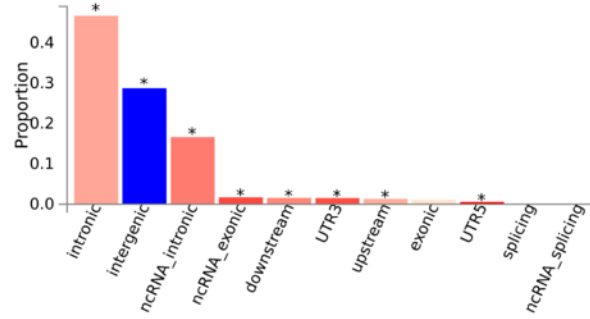

B

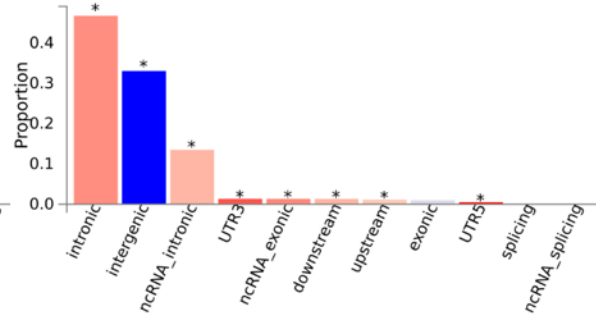

C

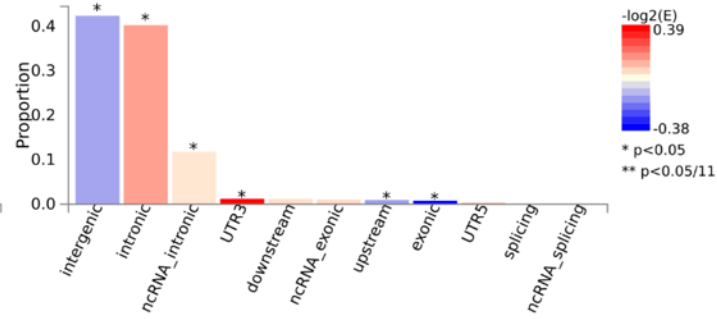

D

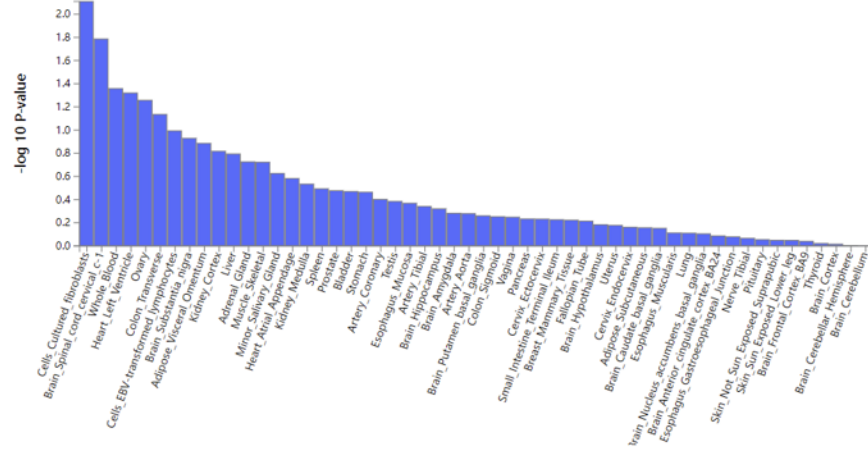

E

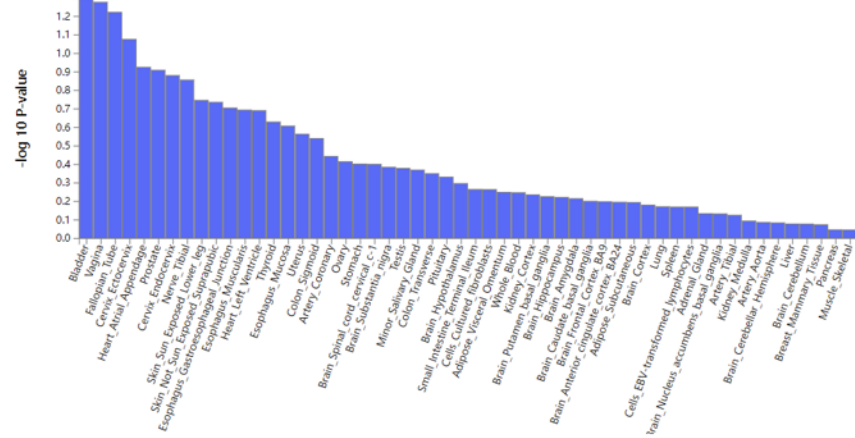

Supplement: Supplementary file 1 [file biology-11-01345-s001.zip › Figure S4.pdf]

A

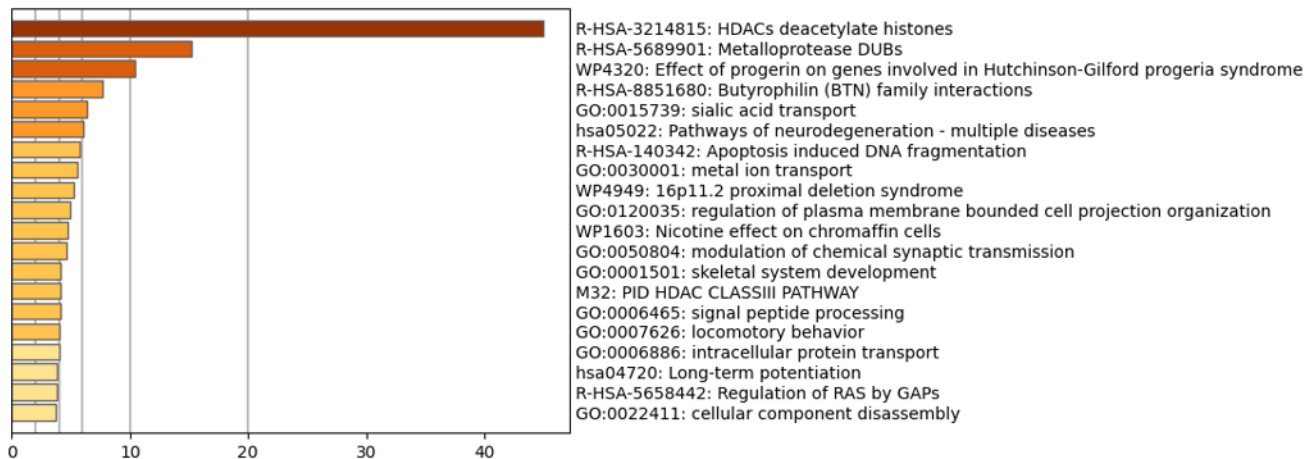

B

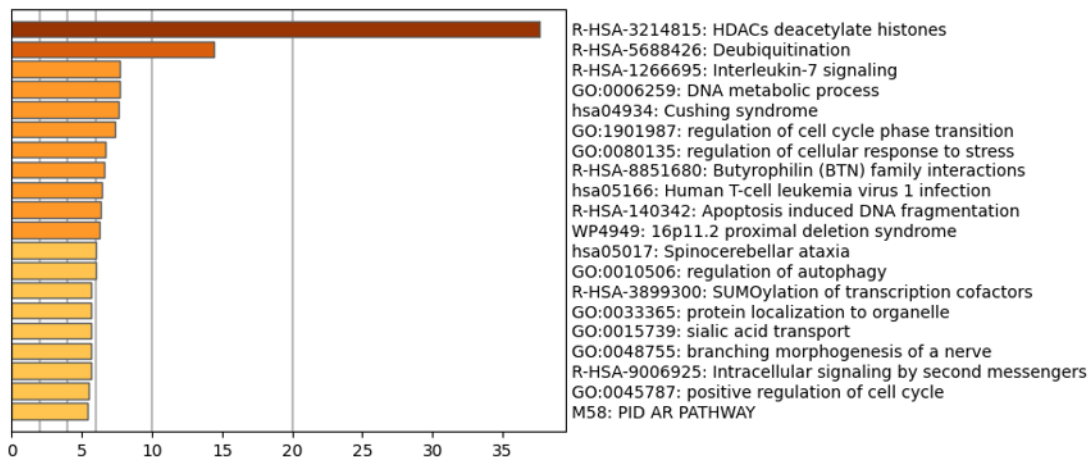

C

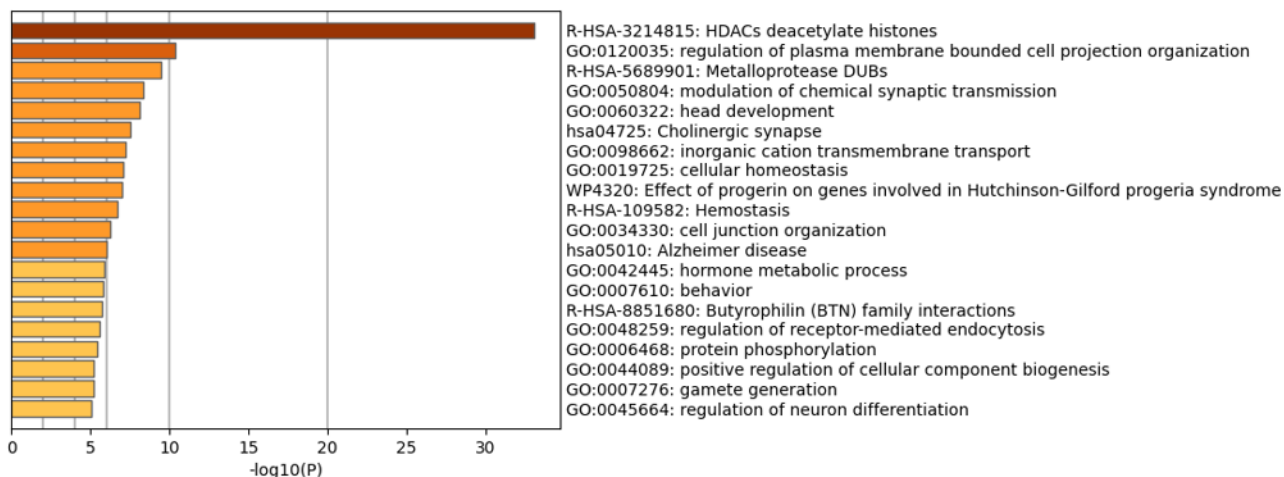

Supplement: Supplementary file 1 [file biology-11-01345-s001.zip › Figure S5.pdf]
